# The validity and reliability of the OneStep smartphone application under various gait conditions in healthy adults with feasibility in clinical practice

**DOI:** 10.1186/s13018-022-03300-4

**Published:** 2022-09-14

**Authors:** Jesse C. Christensen, Ethan C. Stanley, Evan G. Oro, Hunter B. Carlson, Yuval Y. Naveh, Rotem Shalita, Levi S. Teitz

**Affiliations:** 1 Department of Physical Medicine and Rehabilitation, Veterans Affairs Health Care System, Salt Lake City, UT USA; 2 Department of Physical Therapy and Athletic Training, University of Utah, Salt Lake City, UT USA; 3 Celloscope Ltd, Tel Aviv, Israel

**Keywords:** Validity, Reliability, Agreement, Feasibility, Smartphone application, Motion analysis system

## Abstract

**Objective:**

Primary purpose of this study was to determine the validity and reliability of the OneStep smartphone application in healthy adults. Secondary purpose was to determine the feasibility of measuring gait dysfunction, limitation in spatiotemporal characteristics, longitudinally in patients following total hip or knee arthroplasty.

**Methods:**

First objective, 20 healthy adults (mean age, 42.3 ± 19.7 years; 60% males; mean body mass index, 29.0 ± 5.2 kg/m^2^) underwent gait analysis under four gait conditions (self-selected gait speed, fixed gait speed at 0.8 m/s, fixed gait speed at 2.0 m/s and self-selected gait speed with dual task) for the validity and reliability of the smartphone to the motion laboratory. Reliability was determined by intraclass correlation coefficients. Validity was determined by Pearson correlations. Agreement was assessed by the Bland–Altman method. Second objective, 12 additional patients with total hip or knee arthroplasty (mean age, 58.7 ± 6.5 years; 58% males; mean body mass index, 28.9 ± 5.8 kg/m^2^) were measured at 2- and 10 weeks postoperatively. The smartphone application was used to evaluate change in gait dysfunction over time within the patients’ own environment using paired *t* test.

**Results:**

The smartphone application demonstrated moderate-to-excellent intraclass correlation coefficients for reliability between-system (ICC range, 0.56–0.99), -limb (ICC range, 0.62–0.99) and -device (ICC range, 0.61–0.96) for gait analysis of healthy adults. Pearson correlations were low-to-very high between methods (*r* range, 0.45–0.99). Bland–Altman analysis revealed relative underestimation of spatiotemporal variables by the smartphone application compared to the motion system. For patients following total hip or knee arthroplasty, gait analysis using the OneStep application demonstrated significant improvement (*p* < 0.001, Cohen’s *d* > 0.95) in gait dysfunction between 2- and 10 weeks postoperatively.

**Conclusion:**

The smartphone application can be a valid, reliable and feasible alternative to motion laboratories in evaluating deficits in gait dysfunction in various environments and clinical settings.

## Introduction

Gait dysfunction is common in older adults [1] and associated with greater decline in physical function [2, 3]. Adults with gait dysfunction suffer losses of independence, diminished ability to perform daily activities and can struggle to take part in community events [4, 5]. Older adults experience varying gait conditions during daily activities, which require change in speed and cognition in negotiating these environments [1, 5]. Moreover, gait dysfunction in elderly populations often results in falls and injuries, beginning a vicious cycle of functional deterioration [6]. Identifying gait dysfunction is a primary objective in rehabilitation, particularly in populations with joint impairment such as post-total hip (THA) and knee arthroplasty (TKA) [7, 8].

Motion capture analysis is the gold standard for evaluating gait dysfunction in pathological populations [9]. Identifying gait dysfunction, defined as spatiotemporal variables of walking, requires expensive equipment and is largely accessible only in motion laboratories, thus direct translation to clinical practice is limited. More importantly, the motion laboratory does not reflect natural environments, providing a potentially biased assessment of gait dysfunction. Alternatively, clinicians will obtain spatiotemporal data using portable pressure walkways or attempt to measure these gait characteristics manually based on visual observation. Unfortunately, these methods can also be expense and unreliable. Thus, using innovative wearable technology may provide a more practical means of detecting gait dysfunction in adults pre- and post-surgery.

Smartphone applications might be a novel solution to provide accurate, affordable and practical means of real-time information to clinicians working to identify gait dysfunction. Validation of smartphone applications are currently restricted to walking within limited gait conditions and are largely implemented within young adults [10, 11]. These limitations in smartphone-derived validity make the translation to natural environments and older pathological populations challenging. It is essential to evaluate gait variables under conditions that older adults are likely to encounter in daily activity. To date, many smartphone applications have been compared to the gold standard motion laboratory to determine basic spatiotemporal variables (e.g., cadence, speed, stride length) of walking [11–14]. However, no study has compared gait parameters beyond the basic spatiotemporal variables, including stance, step length, single and double support in various gait conditions, leaving questions to the validity and reliability of the product. Furthermore, no study has gone beyond the validation and reliability of the product, and determined the feasibility of incorporating smartphone application into rehabilitation assessment of patients with THA or TKA outside the laboratory. Restoring the ability to walk is a primary goal post-THA or TKA. However, patients post-THA and TKA continue to demonstrate gait dysfunction, reduced single limb stance time, cadence, stride length and gait speed [15, 16], despite proper kinematic alignment and improved self-report outcomes. Therefore, it is clinically relevant to provide clinicians with valid and reliable tools to identify and treat gait dysfunction that do not require a robust motion laboratory.

Therefore, the primary purpose of this study was to determine the validity and reliability of the OneStep smartphone application (Celloscope Ltd., Tel Aviv, Israel) compared to the gold standard Vicon motion analysis system (Vicon, Oxford Metrics Ltd., Oxford, UK) in healthy adults during various gait conditions. The secondary purpose of this study was to determine the feasibility of measuring change in gait characteristics longitudinally in patients with THA or TKA. Our primary hypothesis was a smartphone application evaluation of spatiotemporal variables would be valid and reliable across various gait conditions. We further hypothesized that a smartphone application would be able to monitor improvement in gait dysfunction in patients with THA or TKA when negotiating various walking conditions in their own natural environment.

## Materials and methods

### Participants

The primary study design was cross-sectional with a cohort of healthy adults. The secondary study design was prospective cohort design of patients with THA or TKA. For the primary purpose of the study, an inclusion criterion of adults ≥ 18 years of age that are healthy with no history of orthopedic, neurological, cardiovascular and integumental pathology. Participants were excluded if they had any health condition that may affect their walking or balance abilities. For the secondary purpose of the study, an inclusion criterion of adults between 45 and 70 years of age that successfully underwent an uncomplicated unilateral THA or TKA. Uncomplicated unilateral THA or TKA was defined as patients that did not acquire any postoperative complications such as wound complication, thromboembolic disease, dislocation/instability, periprosthetic fracture, deep periprosthetic joint infection or arthrofibrosis resulting in readmission or revision surgery. Participants were excluded if they had any complications or other unstable orthopedic conditions that limited walking ability. All procedures were approved by the University of Utah Institutional Review Board (IRB#00138995) and all participants provided written, informed consent prior to participating in the study. The precision approach was used for sample size determination [17, 18]. Using the sample size determination approach described by Bonett [19], and assuming intraclass correlation coefficients (ICC) = 0.80, this sample size (*n* = 20) and number of raters (single rater) provides a 95% confidence interval around ICC of width 0.37 (ICC ± 0.18). This seems to be acceptable precision for our purposes, so the sample size and number of raters are adequate.

### Protocol

For the primary purpose of the study, testing was completed at the Motion Capture Core Facility at the University of Utah, Department of Physical Therapy and Athletic Training. Motion analysis was performed using a 10-camera motion analysis system sampling at 200 Hz (Vicon Motion Systems; Oxford, UK). Kinetic data were obtained using a dual-belt instrumented treadmill. (Bertec; Columbus, OH, USA) sampling at 1000 Hz. Spatiotemporal data were recorded and synchronized using Nexus v2.9.3 software (Vicon, Oxford Metrics Ltd., Oxford, UK).

Each participant was fitted with compressive clothing and instrumented with 53 retro-reflective markers (39 markers, 14 mm; 14 markers, 9.5 mm), allowing for tracking of eight body segments. Prior to data collection, the motion analysis system was calibrated, and a standing calibration trial was obtained to determine joint centers and to create a segment coordinate system. The modified Plug-In-Gait marker set (Vicon, Oxford Metrics Ltd., Oxford, UK) defined one HAT segment (combined head, arms and trunk), one pelvis segment, two thigh segments, two shank segments and two foot segments. Marker locations were used for attributing coordinate systems for each segment and were positioned on the seventh cervical spinous process, manubrium of the sternum, inferior body of the sternum, bilaterally on the anterior/posterior superior iliac spines, right spine of scapula, iliac crests, greater trochanters, acromions, medial and lateral epicondyles of the femurs, medial and lateral malleoli, first and fifth heads of the metatarsals, dorsum of the feet and calcaneal tuberosities. Two non-rigid clusters with four non-collinear markers were placed at the lateral side of each thigh and shank. Two smartphones (iPhone SE, Apple Inc., Cupertino, CA, USA) were also affixed to the right and left anterolateral thighs of each participant. The OneStep smartphone application (Celloscope, Tel Aviv, Israel) was downloaded and activated on each device prior to formal data collection. The OneStep smartphone application uses data collected by the smartphone's built-in sensors to measure gait parameters. Acceleration and angular data are collected by the smartphone's sensors at a sampling rate of 100 Hz, and that data are analyzed by proprietary algorithms to measure the spatiotemporal variables for each stride. For each walking trial recorded, the “record walk” button within the application was pressed simultaneously for two smartphones prior to the participant beginning that walk. A single research physical therapist (JCC) donned all markers to each participant in the study. This was implemented to reduce risk of marker placement error across the cohort.

Participants completed four gait conditions: (1) self-selected gait speed, (2) fixed gait speed at 0.8 m/s, (3) fixed gait speed at 2.0 m/s and (4) self-selected gait speed with dual task. The self-selected gait speed was acquired using the 5-m walk test [20]. Participants were instructed to walk at a normal speed and testing used a standard stopwatch, a standard 10-m measured walkway and two marked areas 5 m apart. Three separate trials of the 5-m walk test were completed, and the average for the self-selected gait speed trials was used for formal data collection. The fixed gait speeds constrained at 0.8 and 2.0 m/s were used to provide a slow and fast walking environment on the treadmill. The gait speed of 0.8 m/s was selected as this is a speed that is predictive of poor clinical outcomes [21]. The speed of 2.0 m/s was selected as this is a speed used to determine limits of dynamic stability in older adults [22]. The self-selected gait speed with dual task was defined as ambulating at the designated self-selected speed while performing the *serial seven* test, requiring the participants to count down from 100 backward by sevens. This test is a standard test for evaluating cognition, attention and mental status [23], and was used to provide a cognitive challenge to the participant during the gait trial. Participants completed the non-randomized battery of gait conditions (time 1) and then completed the same battery of gait conditions approximately 60 min later (time 2) to evaluate test–retest reliability.

Prior to each gait condition collection, a 1–2-min warm-up period was provided. Once participants confirmed they felt comfortable with the gait condition, they were asked to walk as naturally as possible. Participants were provided a 5- to 10-min rest period prior to beginning the next gait condition to minimize risk of fatigue. Rating of perceived exertion and numeric pain rating scale were also recorded following completion of each session. A trial, defined as 15 successful steps on each limb, was considered acceptable if all markers were visible and the participant’s foot landed successfully on the instrumented treadmill force platforms without any disturbance to their gait. Trials in which participants lost their balance were excluded. For each walking condition, the first 15 successful steps on each limb were averaged and used for statistical analysis.

*Data processing and analysis* For the first objective, post processing and extraction of spatiotemporal variables were acquired using Nexus software v2.9.3 (Vicon, Oxford Metrics Ltd., Oxford, UK). Spatiotemporal variables of double limb stance time (seconds), single limb stance time (seconds), step length (meters), cadence (steps/minute), stride length (meters), swing time (seconds) and gait speed (meters/second) were computed and compared using OneStep smartphone application and Vicon motion analysis system. An independent researcher (ECS), blinded to the extent of the study, identified all gait events and characteristics for analysis.

For the secondary purpose of the study, data were collected from patients that had undergone an uncomplicated unilateral THA or TKA. Patients with THA or TKA who had undergone remote rehabilitation collected self-recorded walks trials at both 2- and 10 weeks postoperatively in their own environment. The walking trials were 30 sec bouts of continuous walking, while using the smartphone application. The patients were instructed to walk in a straight line as much as possible for 30 sec. Patients were allowed to make turns as needed to walk in a continuous loop if their indoor environment did not allow for continuous walking in a straight line for 30 sec. All gait cycles involved in turning were excluded from the final analysis. Two weeks postoperatively was identified as an appropriate timeframe when most patients with THA or TKA have weaned off assistive devices and beginning to walk independently [24, 25]. Ten weeks postoperatively was identified as most of the physical recovery of walking is obtained by this timeframe [24, 25].

*Data processing and analysis* For the second objective, post-processing and extraction of spatiotemporal variables were acquired using OneStep software version 2.10.4 (Celloscope Ltd., Tel Aviv, Israel). Spatiotemporal variables of double limb stance time (seconds), single limb stance time (seconds), step length (meters), cadence (steps/minute), stride length (meters), swing time (seconds) and gait speed (meters/second) were computed and compared over the 2- and 10-week timepoints using OneStep smartphone application. Two senior researchers (YYN, LST) processed and exported all gait characteristics for analysis.

### Statistical analysis

Descriptive statistics were used to provide demographic characteristics of the participants in this study. For the primary purpose of the study, Bland–Altman plots [26], including 95% tolerance intervals (estimated as 1.96 times the standard deviation of the differences) [27], were generated to analyze and visualize agreement between the smartphone application and motion analysis system for each spatiotemporal variable within each gait condition. This specific form of a 95% tolerance interval, which Bland and Altman called the limits of agreement, assumes that the participant paired differences of the two methods are normally distributed. Thus, the mean value ± 1.96 (standard deviation) is the boundary of the middle 95% of the differences between methods. A ratio of the smartphone and motion analysis system gait speed variables for the fixed gait conditions were used for each participant, and calculated limits of agreement were based on the mean value ± 1.96 (standard deviation). This approach was used as per the recommendations of Bland and Altman et al. [27] as these data were proportional instead of constant across the *x*-axis. Inter- and intra-reliability were quantified by ICCs. Inter-system reliability was measured between smartphone application and motion analysis system. Inter-limb reliability was measured between the smartphone devices secured onto the left and right limbs. Test–retest intra-device reliability was measured between the smartphone application device on the right thigh between time 1 and time 2. Additionally, Pearson correlations were calculated to examine construct validity with the smartphone application as the independent variable and the motion analysis system as the dependent variable. Classification of the strength of the reliability according to ICCs were as follows: poor reliability (ICCs < 0.50), moderate reliability (ICCs = 0.50–0.75), good reliability (ICCs = 0.75–0.90) and excellent reliability (ICCs > 0.90) [28]. Classification of the strength of the association according to Pearson correlations was as follows: negligible (*r* = 0.0–0.3), low (*r* = 0.3–0.5), moderate (*r* = 0.5–0.7), high (*r* = 0.7–0.9), very high (*r* = 0.9–1.0) [29]. For the second purpose of the study, change scores were calculated by taking the difference between the spatiotemporal variables at the approximately 2- and 10-week postoperative timepoints following THA or TKA. Paired *t* tests were used to compare differences in spatiotemporal variables over time. Effect sizes were computed as an indicator of the quantitative strength of the standardized mean differences (Cohen’s *d*) [30]: 0.20 indicates a small effect, equal to or greater than 0.50 indicates a medium effect and equal to or greater than 0.80 indicates a strong effect. Analyses were performed using STATA v17.0 statistical software package (College Station, TX, USA).

## Results

**Fig. 1:**
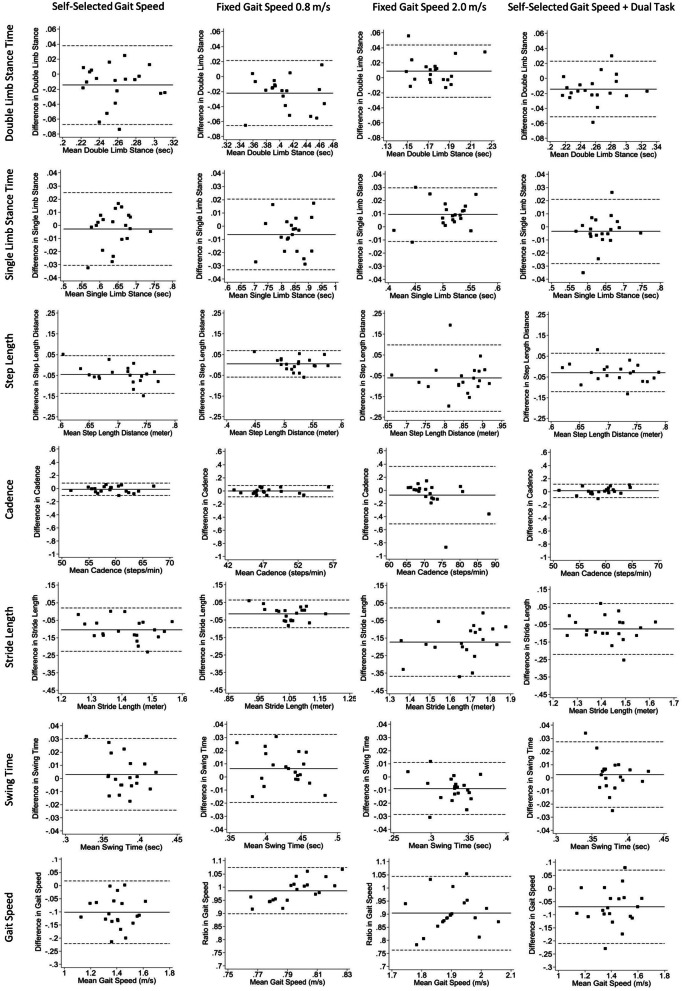
Bland-Altman plots comparing the smartphone application and motion capture system measurements in assessing spatiotemporal variables for each gait condition. Mean bias is displayed as a solid line and 95% limits of agreement are displayed as dashed lines

For the primary purpose of the study, a total of 32 people was screened for enrollment in the study; seven declined and five were excluded due to history of orthopedic surgery (4) and health issues affecting walking and balance ability (1). Therefore, a total of 20 healthy participants were enrolled for the primary purpose of the study (Table 1). For the secondary purpose of the study, a total of 12 patients (mean age, 58.7 ± 6.5 years; 58% males; mean body mass index, 28.9 ± 5.8 kg/m^2^) with an uncomplicated unilateral total joint arthroplasty (hip = 9; knee = 3) were prospectively monitored from approximately 2 weeks (mean, 15.3 ± 2.9 days) and 10 weeks (mean, 74.1 ± 8.2 days) following surgery. Bland–Altman analyses revealed agreement between the smartphone application and motion analysis system as shown in Fig. 1. The results reflected a trend of relatively small underestimation of the smartphone application compared to the motion analysis system across the parameters (Table 2). For time-based parameters (double limb stance time, single limb stance time, and swing time), the mean bias of pooled gait conditions ranged from − 0.01 to 0.001 s (95% limits of agreement: lower limits, − 0.058 to − 0.026; upper limits, 0.026 to 0.037). For distance-based parameters (step length and stride length), mean bias ranged from − 0.091 to − 0.032 m (95% limits of agreement: lower limits, − 0.271 to − 0.149; upper limits, 0.083 to 0.088). Mean bias for cadence was − 0.019 steps/minute (95% limits of agreement: lower, − 0.258; upper, 0.218), and mean bias for gait speed was − 0.095 m/s (95% limits of agreement: lower, − 0.312; upper, 0.122).

**Table 1:** Descriptive characteristics

Variable	Cohort (*n* = 20)
Age, y	42.3 (19.6)
Sex, % male	60
Weight, kg	77.0 (17.4)
Height, m	1.63 (0.24)
BMI, kg/m^2^	29.0 (5.2)
UCLA activity scale	8 (4–10)
CCI	0.85 (0.93)

Values represented as mean (SD), unless otherwise stated. Values for UCLA activity scale represented as mean (range)

*BMI* body mass index, *UCLA* University of California Los Angeles, *CCI* Charlson Comorbidity Index

**Table 2:** Bland–Altman agreement results of spatiotemporal variables between motion capture system and smartphone application

Gait conditions	Mean bias	95% CI mean bias	Upper limit agreement	95% CI upper limit agreement	Lower limit agreement	95% CI lower limit agreement
*Pooled gait conditions*						
Double limb stance time	− 0.010	− 0.015, − 0.005	0.037	0.028, 0.046	− 0.058	− 0.067, − 0.049
Single limb stance time	− 0.001	− 0.003, 0.002	0.026	0.021, 0.031	− 0.028	− 0.033, − 0.022
Step length	− 0.032	− 0.045, − 0.019	0.083	0.061, 0.106	− 0.149	− 0.172, − 0.127
Cadence	− 0.019	− 0.046, 0.006	0.218	0.172, 0.264	− 0.258	− 0.304, − 0.212
Stride length	− 0.091	− 0.111, − 0.071	0.088	0.054, 0.123	− 0.271	− 0.306, − 0.236
Swing time	0.001	− 0.002, 0.003	0.027	0.022, 0.032	− 0.026	− 0.031, − 0.020
Gait speed	− 0.095	− 0.433, 0.102	0.122	0.080, 0.164	− 0.312	− 0.354, − 0.270
*Self-selected gait speed*						
Double limb stance time	− 0.014	− 0.026, − 0.002	0.038	0.016, 0.059	− 0.067	− 0.088, − 0.045
Single limb stance time	− 0.002	− 0.009, 0.003	0.024	0.013, 0.036	− 0.030	− 0.041, − 0.019
Step length	− 0.045	− 0.067, − 0.024	0.045	0.008, 0.082	− 0.136	− 0.173, − 0.099
Cadence	− 0.014	− 0.036, 0.008	0.080	0.042, 0.119	− 0.109	− 0.147, − 0.070
Stride length	− 0.104	− 0.133, − 0.075	0.019	− 0.030, 0.069	− 0.227	− 0.277, − 0.177
Swing time	0.003	− 0.003, 0.009	0.030	0.019, 0.041	− 0.024	− 0.035, − 0.013
Gait speed	− 0.102	− 0.129, 0.074	0.016	− 0.031, 0.065	− 0.221	− 0.269, − 0.172
*Fixed gait speed 0.8 m/s*						
Double limb stance time	− 0.021	− 0.032, − 0.011	0.021	0.003, 0.039	− 0.065	− 0.083, − 0.047
Single limb stance time	− 0.006	− 0.012, − 0.000	0.020	0.009, 0.031	− 0.033	− 0.043, − 0.022
Step length	0.004	− 0.010, 0.019	0.068	0.042, 0.094	− 0.059	− 0.085, − 0.033
Cadence	− 0.003	− 0.023, 0.017	0.084	0.048, 0.119	− 0.090	− 0.125, − 0.054
Stride length	− 0.014	− 0.033, 0.003	0.064	0.032, 0.096	− 0.093	− 0.125, − 0.061
Swing time	0.006	0.000, 0.012	0.032	0.021, 0.042	− 0.019	− 0.029, − 0.008
Gait speed	− 0.011	− 0.027, 0.005	0.059	0.030, 0.088	− 0.081	− 0.110, − 0.053
*Fixed gait speed 2.0 m/s*						
Double limb stance time	0.008	0.001, 0.017	0.043	0.029, 0.057	− 0.025	− 0.040, − 0.011
Single limb stance time	0.009	0.004, 0.013	0.029	0.021, 0.037	− 0.011	− 0.019, − 0.002
Step length	− 0.061	− 0.098, − 0.023	0.099	0.034, 0.163	− 0.221	− 0.286, − 0.156
Cadence	− 0.074	− 0.176, 0.028	0.362	0.185, 0.539	− 0.511	− 0.688, − 0.334
Stride length	− 0.172	− 0.218, − 0.126	0.022	− 0.056, 0.102	− 0.367	− 0.446, − 0.288
Swing time	− 0.008	− 0.013, − 0.004	0.010	0.002, 0.018	− 0.028	− 0.036, − 0.020
Gait speed	− 0.196	− 0.245, − 0.147	0.089	− 0.026, 0.205	− 0.483	− 0.599, − 0.367
*Self-selected gait speed and dual task*						
Double limb stance time	− 0.014	− 0.022, − 0.005	0.022	0.007, 0.037	− 0.051	− 0.066, − 0.036
Single limb stance time	− 0.003	− 0.009, 0.002	0.021	0.011, 0.031	− 0.027	− 0.037, − 0.018
Step length	− 0.029	− 0.051, − 0.007	0.063	0.025, 0.100	− 0.121	− 0.159, − 0.084
Cadence	0.012	− 0.011, 0.036	0.116	0.074, 0.158	− 0.091	− 0.133, − 0.049
Stride length	− 0.074	− 0.108, − 0.040	0.070	0.011, 0.129	− 0.220	− 0.279, − 0.161
Swing time	0.002	− 0.003, 0.008	0.027	0.017, 0.037	− 0.022	− 0.032, − 0.012
Gait speed	− 0.070	− 0.102, − 0.037	0.069	0.012, 0.126	− 0.210	− 0.266, − 0.153

A positive mean bias value indicates the smartphone application (OneStep) overestimated the variable compared to the motion capture system (Vicon). A negative mean bias value indicates the smartphone application underestimated the variable compared to the motion capture system

Pearson correlations between the smartphone application and motion analysis system across all spatiotemporal variables ranged from low to very high (range, *r* = 0.45–0.99; Table 3). For the pooled gait conditions, the agreement results across spatiotemporal variables were very high (range, *r* = 0.91–0.99; Table 3). For the self-selected gait speed trial, the agreement results across spatiotemporal variables ranged from moderate to very high (range, *r* = 0.62–0.99; Table 3). For the fixed gait speed at 0.8 m/s trial, the agreement results across spatiotemporal variables ranged from moderate to very high (range, *r* = 0.58–0.99; Table 3). For the fixed gait speed at 2.0 m/s trial, the agreement results across spatiotemporal variables ranged from low to very high (range, *r* = 0.45–0.99; Table 3). For the self-selected gait speed trial with dual task, the agreement results across spatiotemporal variables ranged from moderate to very high (range, *r* = 0.63–0.99; Table 3).

**Table 3:** Spatiotemporal comparison between motion capture system and smartphone application

Gait conditions	System	Mean, SD	*r*^ꞙ^
*Pooled gait conditions*			
Double limb stance time (s)	Vicon	0.279, 0.094	0.96
	OneStep	0.269, 0.084	
Single limb stance time (s)	Vicon	0.656, 0.128	0.99
	OneStep	0.655, 0.124	
Step length (m)	Vicon	0.711, 0.139	0.91
	OneStep	0.678, 0.114	
Cadence (steps/min)	Vicon	59.51, 9.58	0.99
	OneStep	59.49, 9.54	
Stride length (m)	Vicon	1.43, 0.269	0.95
	OneStep	1.34, 0.221	
Swing time (s)	Vicon	0.377, 0.041	0.95
	OneStep	0.378, 0.044	
Gait speed (m/s)	Vicon	1.42, 0.439	0.97
	OneStep	1.32, 0.377	
*Self-selected gait speed*			
Double limb stance time (s)	Vicon	0.264, 0.030	0.62
	OneStep	0.250, 0.029	
Single limb stance time (s)	Vicon	0.641, 0.042	0.95
	OneStep	0.638, 0.045	
Step length (m)	Vicon	0.728, 0.060	0.65
	OneStep	0.682, 0.040	
Cadence (steps/min)	Vicon	59.12, 3.61	0.99
	OneStep	59.11, 3.61	
Stride length (m)	Vicon	1.46, 0.103	0.80
	OneStep	1.36, 0.019	
Swing time (s)	Vicon	0.377, 0.025	0.85
	OneStep	0.380, 0.020	
Gait speed (m/s)	Vicon	1.44, 0.127	0.89
	OneStep	1.34, 0.124	
*Fixed gait speed 0.8 m/s*			
Double limb stance time (s)	Vicon	0.418, 0.037	0.83
	OneStep	0.396, 0.035	
Single limb stance time (s)	Vicon	0.840, 0.055	0.97
	OneStep	0.834, 0.056	
Step length (m)	Vicon	0.518, 0.037	0.58
	OneStep	0.523, 0.030	
Cadence (steps/min)	Vicon	47.63, 3.15	0.99
	OneStep	47.62, 3.16	
Stride length (m)	Vicon	1.05, 0.067	0.81
	OneStep	1.04, 0.054	
Swing time (s)	Vicon	0.423, 0.033	0.92
	OneStep	0.430, 0.029	
Gait speed (m/s)	Vicon	0.800, 0.012	0.85
	OneStep	0.789, 0.032	
*Fixed gait speed 2.0 m/s*			
Double limb stance time (s)	Vicon	0.171, 0.020	0.63
	OneStep	0.179, 0.020	
Single limb stance time (s)	Vicon	0.503, 0.037	0.97
	OneStep	0.513, 0.039	
Step length (m)	Vicon	0.870, 0.076	0.45
	OneStep	0.829, 0.076	
Cadence (steps/min)	Vicon	72.10, 5.64	0.99
	OneStep	72.03, 5.55	
Stride length (m)	Vicon	1.75, 0.134	0.81
	OneStep	1.57, 0.165	
Swing time (s)	Vicon	0.332, 0.024	0.92
	OneStep	0.324, 0.022	
Gait speed (m/s)	Vicon	2.00, 0.102	0.71
	OneStep	1.80, 0.109	
*Self-selected gait speed and dual task*			
Double limb stance time (s)	Vicon	0.264, 0.030	0.83
	OneStep	0.250, 0.032	
Single limb stance time (s)	Vicon	0.639, 0.040	0.96
	OneStep	0.636, 0.044	
Step length (m)	Vicon	0.728, 0.058	0.63
	OneStep	0.698, 0.047	
Cadence (steps/min)	Vicon	59.18, 3.31	0.99
	OneStep	59.20, 3.33	
Stride length (m)	Vicon	1.46, 0.108	0.76
	OneStep	1.39, 0.100	
Swing time (s)	Vicon	0.377, 0.023	0.85
	OneStep	0.379, 0.020	
Gait speed (m/s)	Vicon	1.44, 0.133	0.87
	OneStep	1.37, 0.140	

Standard deviation (SD), Pearson correlation coefficient (*r*)

^ꞙ^< 0.30 (negligible), 0.30–0.50 (low), 0.50–0.70 (moderate), 0.70–0.90 (high), > 0.90 (very high)

The pooled data across all gait conditions were excellent for the inter-system reliability (range, ICC = 0.90–0.99; Table 4), the inter-limb reliability (range, ICC = 0.93–0.99; Table 4) and test–retest intra-device reliability (range, 0.94–0.98; Table 4). The individual gait trials (self-selected gait speed, fixed gait speed and self-selected gait speed trial with dual task) demonstrated inter-system reliability ranging from moderate to excellent (range, ICC = 0.56–0.99; Table 4), the inter-limb reliability ranged from moderate to excellent (range, ICC = 0.62–0.99; Table 4) and test–retest intra-device reliability ranged from good to excellent (range, 0.61–0.93; Table 4).

**Table 4:** Inter- and intra-reliability results between-system, -limb and -device

Gait conditions	Inter-system reliability		Inter-limb reliability		Test–retest intra-device reliability	
	ICC^¥^	95% CI	ICC^¥^	95% CI	ICC^¥^	95% CI
*Pooled gait conditions*						
Double limb stance time	0.96	0.94, 0.97	0.99	0.99, 0.99	0.98	0.97, 0.98
Single limb stance time	0.99	0.99, 0.99	0.99	0.98, 0.99	0.98	0.97, 0.98
Step length	0.90	0.84, 0.93	0.94	0.91, 0.96	0.97	0.96, 0.98
Cadence	0.99	0.99, 0.99	0.99	0.99, 0.99	0.98	0.97, 0.98
Stride length	0.93	0.89, 0.95	0.96	0.93, 0.97	0.96	0.94, 0.97
Swing time	0.95	0.92, 0.96	0.93	0.90, 0.95	0.94	0.86, 0.97
Gait speed	0.96	0.94, 0.97	0.99	0.99, 0.99	0.98	0.97, 0.99
*Self-selected gait speed*						
Double limb stance time	0.61	0.25, 0.82	0.82	0.59, 0.92	0.90	0.77, 0.95
Single limb stance time	0.95	0.87, 0.97	0.94	0.85, 0.97	0.91	0.79, 0.96
Step length	0.60	0.23, 0.82	0.72	0.40, 0.87	0.81	0.57, 0.91
Cadence	0.99	0.99, 0.99	0.99	0.99, 0.99	0.93	0.83, 0.97
Stride length	0.79	0.53, 0.91	0.82	0.60, 0.92	0.83	0.62, 0.92
Swing time	0.83	0.62, 0.92	0.80	0.57, 0.91	0.90	0.76, 0.95
Gait speed	0.89	0.73, 0.95	0.90	0.77, 0.96	0.92	0.80, 0.96
*Fixed gait speed 0.8 m/s*						
Double limb stance time	0.82	0.60, 0.92	0.91	0.79, 0.96	0.88	0.72, 0.94
Single limb stance time	0.97	0.92, 0.98	0.94	0.85, 0.97	0.80	0.56, 0.91
Step length	0.56	0.18, 0.80	0.72	0.42, 0.88	0.88	0.72, 0.94
Cadence	0.99	0.99, 0.99	0.99	0.99, 0.99	0.80	0.57, 0.91
Stride length	0.79	0.54, 0.91	0.86	0.67, 0.94	0.88	0.72, 0.95
Swing time	0.92	0.80, 0.96	0.74	0.45, 0.88	0.74	0.46, 0.88
Gait speed	0.76	40, 0.90	0.70	0.38, 0.86	0.61	0.24, 0.82
*Fixed gait speed 2.0 m/s*						
Double limb stance time	0.63	0.27, 0.83	0.72	0.42, 0.88	0.78	0.53, 0.90
Single limb stance time	0.97	0.91, 0.98	0.99	0.97, 0.99	0.86	0.68, 0.94
Step length	0.62	0.04, 0.85	0.71	0.39, 0.87	0.85	0.66, 0.93
Cadence	0.99	0.99, 0.99	0.99	0.99, 0.99	0.89	0.74, 0.95
Stride length	0.79	0.54, 0.91	0.85	0.66, 0.93	0.72	0.41, 0.87
Swing time	0.91	0.79, 0.96	0.97	0.92, 0.98	0.91	0.79, 0.96
Gait speed	0.63	0.27, 0.83	0.62	0.26, 0.83	0.83	0.62, 0.92
*Self-selected gait speed and dual task*						
Double limb stance time	0.82	0.60, 0.92	0.73	0.44, 0.88	0.93	0.84, 0.97
Single limb stance time	0.96	0.89, 0.98	0.95	0.88, 0.98	0.96	0.91, 0.98
Step length	0.62	0.25, 0.83	0.82	0.59, 0.92	0.93	0.84, 0.97
Cadence	0.99	0.99, 0.99	0.99	0.99, 0.99	0.98	0.95, 0.99
Stride length	0.76	0.48, 0.89	0.76	0.49, 0.89	0.97	0.92, 0.98
Swing time	0.84	0.63, 0.93	0.77	0.50, 0.90	0.93	0.84, 0.97
Gait speed	0.87	0.70, 0.94	0.86	0.67, 0.94	0.98	0.96, 0.99

Intraclass correlation coefficient (ICC); confidence interval (CI). Inter-device reliability was measured between motion capture system (Vicon) and smartphone application (OneStep). Inter-limb reliability was measured between smartphone application device secured onto the left and right limbs. Test–retest intra-device reliability was measured between the smartphone application device on the right thigh between time 1 and time 2

^¥^< 0.50, poor reliability; 0.50–0.75, moderate reliability; 0.75–0.90, good reliability; > 0.90, excellent reliability

**Fig. 2:**
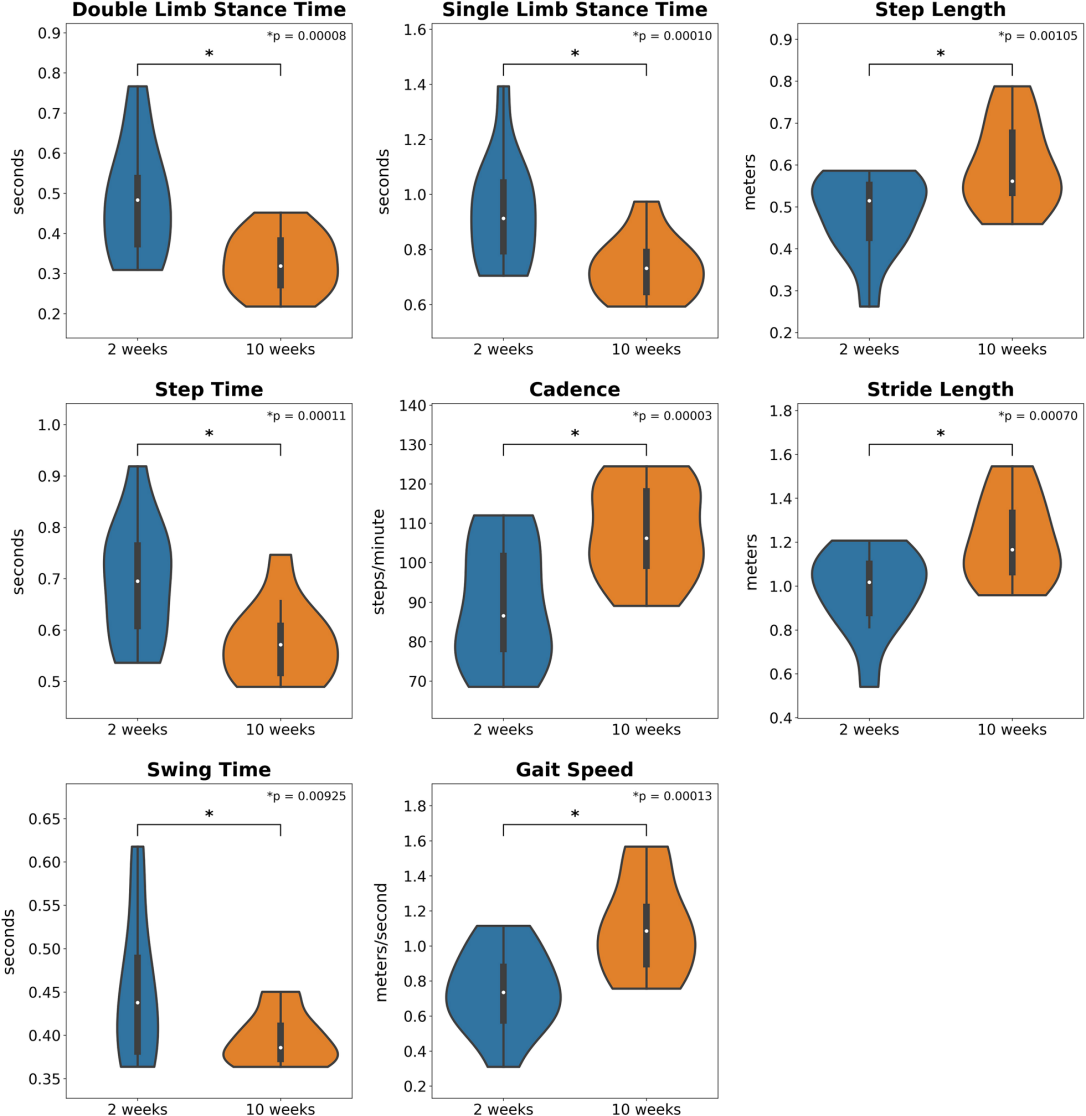
Violin plots comparing the spatiotemporal difference scores between the 2- and 10-week follow-up in adults with total hip and knee arthroplasty. The shape of each violin plot is the kernel density estimation of the data distribution. The median of the distribution is displayed as a white dot, the interquartile range as a thick black bar and the range of outliers as thin black bars within the kernel density estimation

Patients with THA or TKA demonstrated a significant improvement from approximately 2 to 10 weeks (Fig. 2) in double limb stance time [mean difference (MD) =  − 0.153 ± 0.084 s; *p* < 0.01; effect size (ES) =  − 1.82], single limb stance time (MD =  − 0.201 ± 0.113 s; *p* < 0.01; ES =  − 1.77); step length (MD = 0.115 ± 0.086 m; *p* < 0.01; ES = 1.757); cadence (MD = 18.53 ± 9.04 steps/min; *p* < 0.01; ES = 2.05); stride length (MD = 0.233 ± 0.166 m; *p* < 0.01; ES = 1.40); swing time (MD =  − 0.055 ± 0.058 s; *p* < 0.01; ES =  − 0.95) and gait speed (MD = 0.361 ± 0.208 m/s; *p* < 0.01; ES = 1.73).

## Discussion

The primary purpose of this study was to determine the validity and reliability of the OneStep smartphone application compared to the Vicon motion analysis system in healthy adults during various gait conditions. Our secondary purpose of this study was to determine the feasibility of measuring functional recovery in gait dysfunction longitudinally in patients with THA or TKA. The primary findings are as follows: the smartphone application demonstrated low measurement error, large effect sizes and good reliability compared to the motion analysis system for assessing spatiotemporal variables during various gait conditions in healthy adults. The secondary findings showed the smartphone application demonstrated significant and clinically meaningful changes over time in the spatiotemporal variables in patients with THA or TKA following 10 weeks of physical rehabilitation. These data showed it is feasible to implement a smartphone application into postoperative rehabilitation to monitor changes in gait dysfunction following surgery.

To our knowledge, this is the first study to examine validity and reliability of a smartphone application focused on various gait conditions, while also evaluating the feasibility of integrating the device into rehabilitation within a pathological orthopedic population. Our findings are particularly encouraging as the smartphone application showed moderate to very high construct validity to the motion analysis system and moderate to excellent reliability between-system (ICC range, 0.56–0.99), -limb (ICC range, 0.62–0.99) and -device (ICC range, 0.61–0.96). Prior study has shown high correlations comparing alternative smartphone technology to a motion analysis system on step length and cadence during level walking and turns [13]. These investigators also showed slower walking speeds showed negligible to moderate validity with higher rates of bias. Further investigation has shown various smartphone applications report inaccurate data particularly in persons with slower, short or non-stereotypical gait patterns [10]. In our study, the OneStep smartphone application demonstrated moderate to very high correlations on similar spatiotemporal variables with consistent results across relatively slow (0.8 m/s) and fast (2.0 m/s) gait conditions. Similar psychometric metrics were also observed during the cognitively demanding dual task, which provides insight that the smartphone application can be effective in a multitude of environments. However, it is important to note that alternative environments (i.e., non-laboratory conditions) could produce different gait characteristics. With that said, the gait conditions tested in this study have not been studied previously, lending new information on how smartphone applications respond to demands observed during daily activities. Moreover, alternative smartphone technology has shown high to very high validity and good to excellent reliability in assessing spatiotemporal variables across level walking, ambulating over irregular surface and crossing an obstacle [11]. Our data suggest that the smartphone application is a reasonable and clinically feasible substitute to motion laboratories for assessing spatiotemporal variables during adult gait.

Though the smartphone application quantification of spatiotemporal variables were accurate across various gait conditions, it was also important to evaluate the reliability of the device. Previous studies have compared the reliability of the smartphone to the motion analysis system based on various smartphone locations ranging from low back to anterior pelvis [10, 11, 13]. However, these are not practical locations individuals place their smartphone, leading to possible concerns in the accuracy of the results when the location of the device changes. The current study attempted to address this concern by securing the smartphone near the front pant pocket providing a more realistic location during daily activities. Our findings showed moderate-to-excellent (range, ICC = 0.56–0.99) reliability to the motion analysis system in this location. It was also important to show reliability between sides of the limb as individuals have different smartphone location preferences. Our findings showed good-to-excellent (ICC = 0.62–0.99) inter-limb reliability for both left and right limb location, indicating preference of the smartphone did not negatively influence the results. Lastly, the test–retest reliability of smartphone technology has not been thoroughly studied and important to have confidence in the accuracy of the results over time. Our findings indicated moderate-to-excellent (ICC = 0.61–0.96) test–retest reliability over time within each gait condition, but excellent across all gait conditions (ICC = 0.94–0.98). Prior study showed similar findings to ours when comparing test–retest reliability over two weeks for walking speed (ICC = 0.89), cadence (ICC = 0.90) and stride length (ICC = 0.89) in a relatively young cohort post-concussion [31]. It is important to note that double limb stance time, step length, and gait speed showed lower metrics of reliability and validity compared to other parameters, particularly during the faster walking condition. These results may be a consequence of the limited range of values for these parameters in each of the individual gait conditions, which depress the values of measures of correlation. Additionally, walking at 2.0 m/s is a relatively demanding challenge, likely resulting in increased variability in gait mechanics compared to the other gait conditions and could have influenced these results. Notably, the reliability and validity of these parameters in the pooled gait conditions was very high (0.90–0.96), indicating excellent performance in a broader range of parameter values. This is further supported by the limits of agreement for these parameters in both pooled and individual conditions, which show that the system is capable of discriminating between clinically relevant differences of these parameters. These data provide promise that a smartphone application can reliably provide reliable spatiotemporal information between systems, location and over time.

Finally, our data were able to show longitudinal improvement in gait dysfunction during the acute and post-acute period of recovery in patients with THA or TKA. These analyses are useful when considering whether a smartphone application offers an appropriate clinical alternative to the traditional motion laboratories for monitoring gait dysfunction. Gait dysfunction will undergo profound changes during the recovery of a joint disease, particularly post-THA and TKA [8, 32]. These changes are multifaceted as patients experience limitation in ability to ambulate due to knee pain and muscle weakness acutely postoperatively. While improvement in gait dysfunction is generally observed as deficits in joint pain and muscle weakness are resolved [33], compensatory strategies remain for years following surgery [16]. This is important as unresolved gait dysfunction relates to poor functional performance [34], muscle weakness [35] and accelerated arthritis changes in the contralateral limb [36]. Thus, having a more practical methods of identifying gait dysfunction and monitoring psychometric performance over time is important, particularly to healthcare providers that have limited access to motion laboratories. Promotion of walking with use of innovative smartphone technology to correct gait dysfunction should be a focused area of future research to improve long-term health outcomes post-THA or TKA. These findings provide new insight on how cost-effective and unobtrusive smartphone technology can identify gait dysfunction in a non-laboratory clinical setting in patients with THA or TKA during the early stages of recovery.

This study has limitations that need to be considered when interpreting the data. First, our cohort consisted of a range of healthy adults. Further study of participants with different demographics (i.e., body mass, morbidities, etc.) or pathological populations could increase the rigor of these methods. Second, the test–retest time was short, potentially influencing results that were captured over longer periods of time. This length of time was chosen as gait characteristics can change over time, and we wanted to evaluate test–retest within a similar environment to determine reliability. Third, the participants enrolled in the primary objective of the study were not defined as older (≥ 65 years of age), so differences in gait characteristics may be different within an alternative patient population. Fourth, while we provided data in patients with THA or TKA in their own natural environment, it would be valuable to have a larger sample size and longer-term follow-up data postoperatively to determine change over time.

## Conclusions

The OneStep smartphone application was shown to be a valid and reliable tool for assessing spatiotemporal variables across various gait conditions in healthy adults. The smartphone application can also monitor improvement in gait in patients with THA or TKA during the acute and post-acute period of recovery. Along with being accurate, affordable and practical, smartphones can be a useful alternative to motion laboratories in evaluating gait dysfunction in adults’ own environment and clinical settings.

## Data Availability

Not available due to individual privacy.

## Abbreviations

THA: Total hip arthroplasty
TKA: Total knee arthroplasty
ICC: Intraclass correlation coefficients
MD: Mean difference
ES: Effect size
